# Genotypes of Papillary Thyroid Carcinoma With High Lateral Neck Metastasis in Chinese Population

**DOI:** 10.3389/fonc.2022.816897

**Published:** 2022-07-05

**Authors:** Wei Guo, Junwei Huang, Taiping Shi, Hanyuan Duan, Xiaohong Chen, Zhigang Huang

**Affiliations:** ^1^ Department of Otolaryngology Head and Neck Surgery, Beijing Tongren Hospital, Capital Medical University, Beijing, China; ^2^ BGI (Beijing Genomics Institution), BGI-Shenzhen, Shenzhen, China

**Keywords:** papillary thyroid carcinoma (PTC), mutational landscape, head and neck (H&N) cancer, thyroid cancer, driver gene

## Abstract

Papillary Thyroid Carcinoma (PTC) is one of the most commonly diagnosed cancer types in China, characterized by its early age at diagnosis and high 25-year survival rate. Distinct mutational patterns in PTC have been linked to activation of the mitogen-activated protein kinase (MAPK) signaling pathway. To explore the clinical significance of genomic variation patterns in Chinese patients with thyroid carcinoma, we investigated the genomic variants in 83 PTC cases with complete clinical records. The mutational patterns were evaluated using a 688-gene panel which covered known driver genes in PTC tumorigenesis, and featured genetic markers in various PTC-related pathways. We evaluated the relationship between mutational landscape and various clinical information in PTC patients with lateral lymph node metastasis. BRAF V600E was the most common mutation. Mutations in NF1, CDC27, PMS2 and PPP4R2 were more common in men, and mutations in NF1, PMS2 and TERT were related to lateral lymph node metastasis. According to the clustering of mutational patterns, we show that the underline driving mechanisms in lateral lymph node metastasis can be divided into two major groups (BRAF-TERT pathway, and NF1-PMS2 pathway). When combined with the TERT mutations, the BRAF mutation group was prone to lateral lymph node metastasis, particularly in elderly women. The NF1 mutations usually co-existed with PMS2 mutations, and this group included more men and young patients who had a high tumor mutational burden and lateral lymph node metastasis rate.

## Introduction

Papillary thyroid cancer (PTC) is a commonly observed malignancy in the endocrine system. The incidence of thyroid cancer in urban areas of China ranks 4th among all malignant tumors in women, increasing at a rate of 20% per year (1).. Although the early detection rate and long-term survival rate of patients with PTC are both very high, there are still some patients with extensive metastasis while the primary lesion is small (2, 3). Even in microcarcinoma, the disease-specific mortality is 0.5% (4). In the current debate of over-treatment and under-treatment, individualized precise treatment is still a clinical problem to be resolved.

Next-generation sequencing-guided molecular diagnostics can provide useful information for personalized treatment in PTC patients. Studies have shown that genetic changes in several PTC-related signaling pathways are key to the carcinogenic mechanism of thyroid cancer (5, 6). The aim of this study was to use the O-seq 688-gene panel to describe the mutational landscape and the possible underlying driving mechanisms in 83 Chinese PTC cases. Two groups of high-risk PTC patients with lateral lymph node metastasis were screened out.

## Methods

### Patient Recruitment and Consent

In total, 100 consecutive patients diagnosed with primary PTC in Tongren Hospital between 2018 and 2019 were enrolled in this study. All patients underwent thyroidectomy (including partial lobectomy of thyroid and total thyroidectomy) and lymph node lymph node dissection. In 17 patients, there was insufficient tumor tissue for analysis so these patients were excluded from the study. Tumor tissue and blood samples from the other 83 patients were saved for further analysis.

There are 15 histological subtypes of PTC according to the New 2017 World Health Organization Classification. The subtypes involved in this study included classic type, papillary microcarcinoma, tall cell variant, follicular variant and oncocytic variant. All patients involved in this study were clearly informed about its purpose, and understood the study protocol. They all signed an informed consent form and agreed to publication of the results of the study.

### DNA Extraction and Sequencing Workflow

Formalin-fixed paraffin-embedded (FFPE) tissue samples were obtained from each patient and combined probe-anchored polymer sequencing was used to detect genetic mutations. The recommended tumor cell content was not less than 20%. DNA was extracted from FFPE samples using a Nucleic Acid Extraction Kit (BGI, Wuhan, China). The DNA was then fragmented, end repaired, and underwent PCR amplification to obtain a pre-PCR library, which was then subjected to targeted capture using the O-seq gene panel. The captured fragments were PCR amplified for sequencing. Post-PCR products were quantified by Qubit^®^ 2.0 (Life Tech, Invitrogen, USA) using QubitTM dsDNA HS Assay Kits (Life Tech, Invitrogen, USA). Samples with a concentration of ≥4 ng/μL were regarded as being of sufficient quality for further analysis. The DNBs were loaded onto chips and sequenced on the BGISEQ-500 sequencing platform (BGI, Shenzhen, China) for targeted sequencing in accordance with the manufacturer’s instructions.

### Data Preparation

Low quality reads were removed from sequencing data using SOAPnuke v1.2.0, after which the clean sequencing data were mapped to the human genome (hg19) using BWA aligner v0.6.2 and Samtools v0.1.19. PCR duplicate reads were marked using Picard v1.98. Alignment refinement was performed using GATK v2.3-9.

### SNV Calling

Candidate SNVs were called using a Bayesian Model, and Fisher’s Test and Kolmogorov-Smirnov Test were used to filter the biased SNVs. SNVs were then scored according to GC content, adjacent SNVs and InDels, multiple mapping locations, etc. Finally, SNVs with low score were removed.

### InDel Calling

Candidate InDels were called using local assembly by the de Bruijn approach and which were removed from the local control set. The InDels in simple repeat regions of the human genome were checked again, because of the high probability of sequencing errors.

### Structural Variant (SV Calling)

The clipped parts were collected and mapped to the genome and the locations of the clipped and remaining parts were clustered to determine the accurate break points of the SVs.

### CNV Calling

After GC bias normalization and batch normalization, regions were clustered according to the sequencing depths and CNVs were identified. The B allele frequency was used to validate CNVs.

### TMB Calculation

The total number of both synonymous and non-synonymous SNVs and InDels with maximum allele frequency greater than 1.5% were counted for each sample. Known driver genes were removed from the total count and the count was divided by a panel size of 2.79 to obtain the final TMB value.

### Statistical Analysis

All statistical analysis was conducted using R version 4.1.0 (R Core Team, 2013). The statistical tools employed in this study included the Student’s t-test and Fisher’s exact test. P < 0.05 was considered to indicate a statistically significant difference between groups.

## Results

### Clinical Information

Among the 83 PTC patients, 80 were diagnosed at an early-stage (stage I or II), and 3 were diagnosed at a late-stage (stage III or IV), according to the 2017 American Joint Committee on Cancer (AJCC) TNM system. Judging from the sex composition of the cohort (56 female vs 27 male), women have a significantly higher PTC population incidence than men. The median age at diagnosis for women was 9.8 years earlier than for men patients (p<0.05) (**Table 1**).

**Table 1:** Demographics for the 83 Chinese patients with PTC in this study.

	Female (n = 56)	Male (n = 27)	Tatal (n = 83)	P value
Age [mean (SD)], %	49.80 (12.26)	40.00 (14.14)	46.61 (13.62)	0.002
Clinical stage (number)				1
I-II	54	26	80	
III-IV	2	1	3	
T stage (number)				0.619
T1	33	13	46	
T2	5	5	10	
T3	16	8	24	
T4	2	1	3	
N stage (number)				0.091
N0	24	5	29	
N1a	17	11	28	
N1b	15	11	26	

### Mutational Patterns in PTC

**Figure 1:**
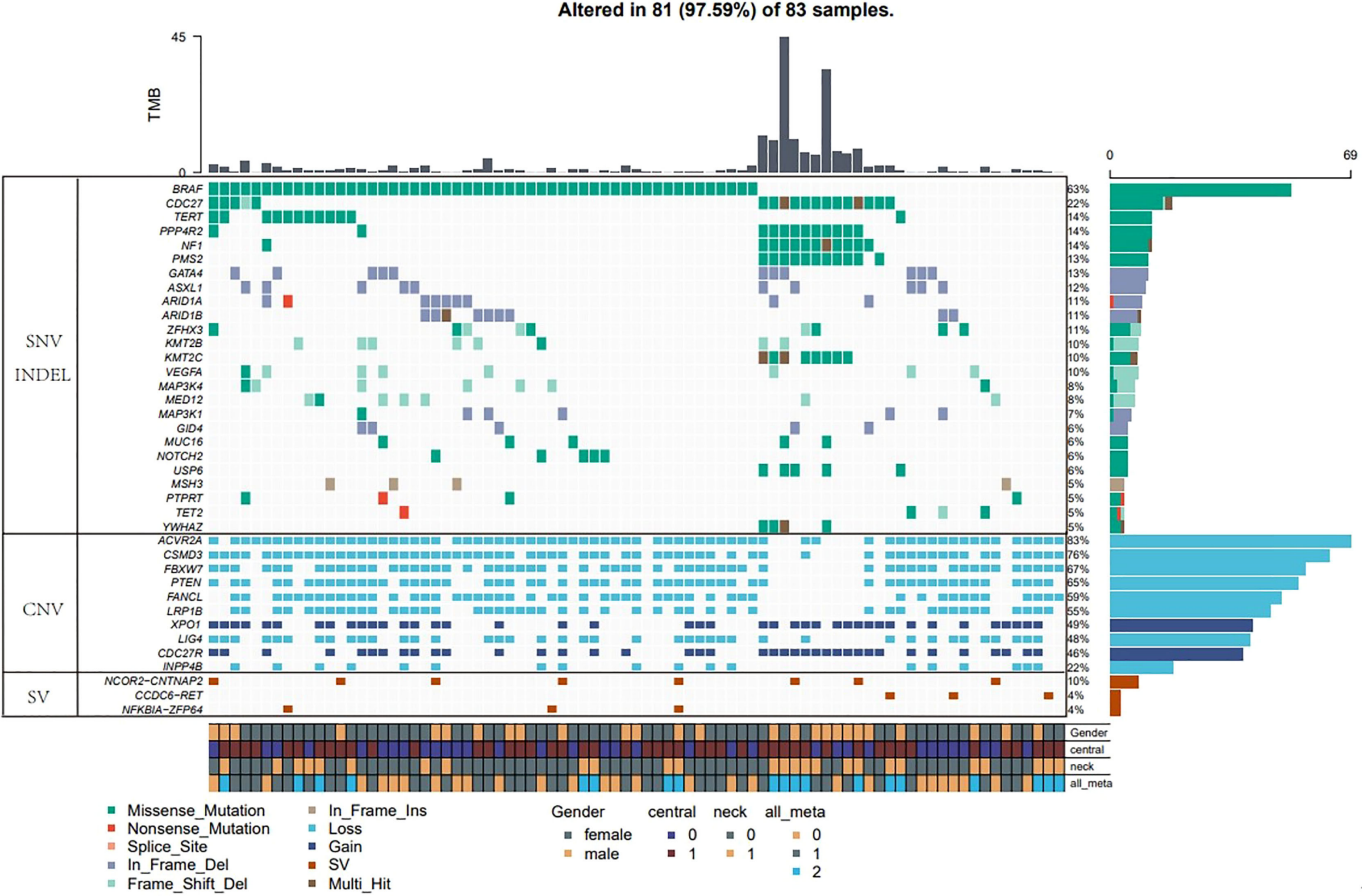
Major types of somatic mutations in 688 genes were investigated in the 83 PTC patients. Landscape of mutation profiles in the 83 PTC patients. Mutation information of each gene in each sample was shown in the waterfall plot, in which various colors with annotations at the bottom represented the different mutation types. The barplot above the legend exhibited the mutation burden. The genes with the highest mutation incidences were BRAF, CDC27, NF1, PPP4R2, TERT and PMS2, corresponding to population percentages of 63%, 22%, 14%, 14%, 14% and 13%, respectively.

Four major types of somatic mutations in 688 genes were investigated in the 83 PTC patients including SNV, Insertions/deletions, gene fusions and CNV. The average number of mutated genes was 16.73 per sample, with male patients on average harboring 3.5 more mutations than female patients. The highest incidence of gene fusion was NCOR2-CNTNAP2, and the population percentage was 10%, followed by the fusion of CCDC6-RET and NFKB1A-ZFP64 with a population percentages of 4% (**Figure 1**).

**Figure 2:**
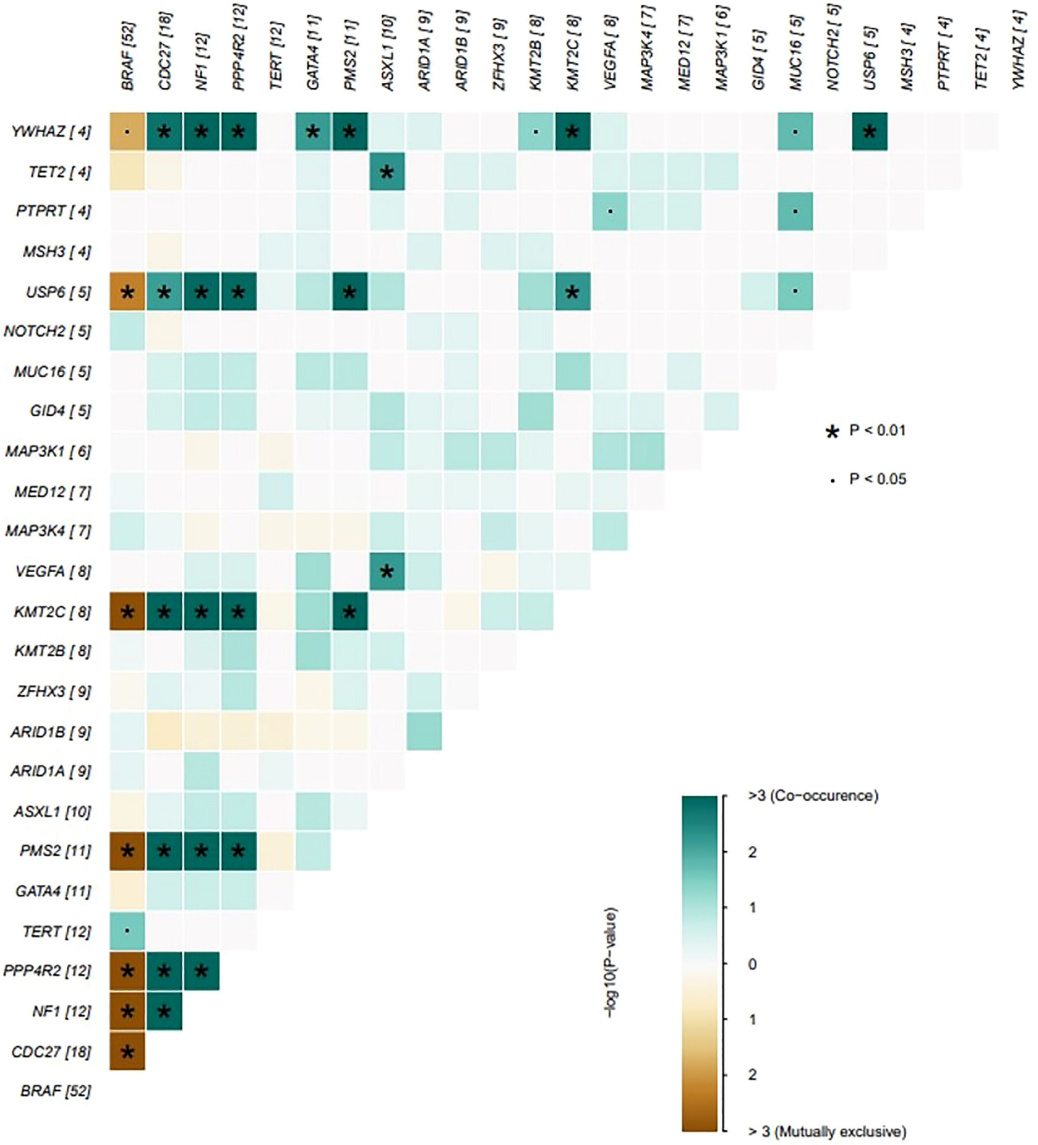
The coincident and exclusive associations across mutated genes. The occurrences of BRAF and NF1/PSM2 mutations were mostly mutually exclusive, *P < 0.01; ^.^ *P* < 0.05.

The genes with the highest mutation incidences were BRAF, CDC27, NF1, PPP4R2, TERT and PMS2, corresponding to population percentages of 63%, 22%, 14%, 14%, 14% and 13%, respectively. The occurrences of BRAF and NF1/PMS2 mutations were mostly mutually exclusive, p<0.01 (**Figure 2**).

**Figure 3:**
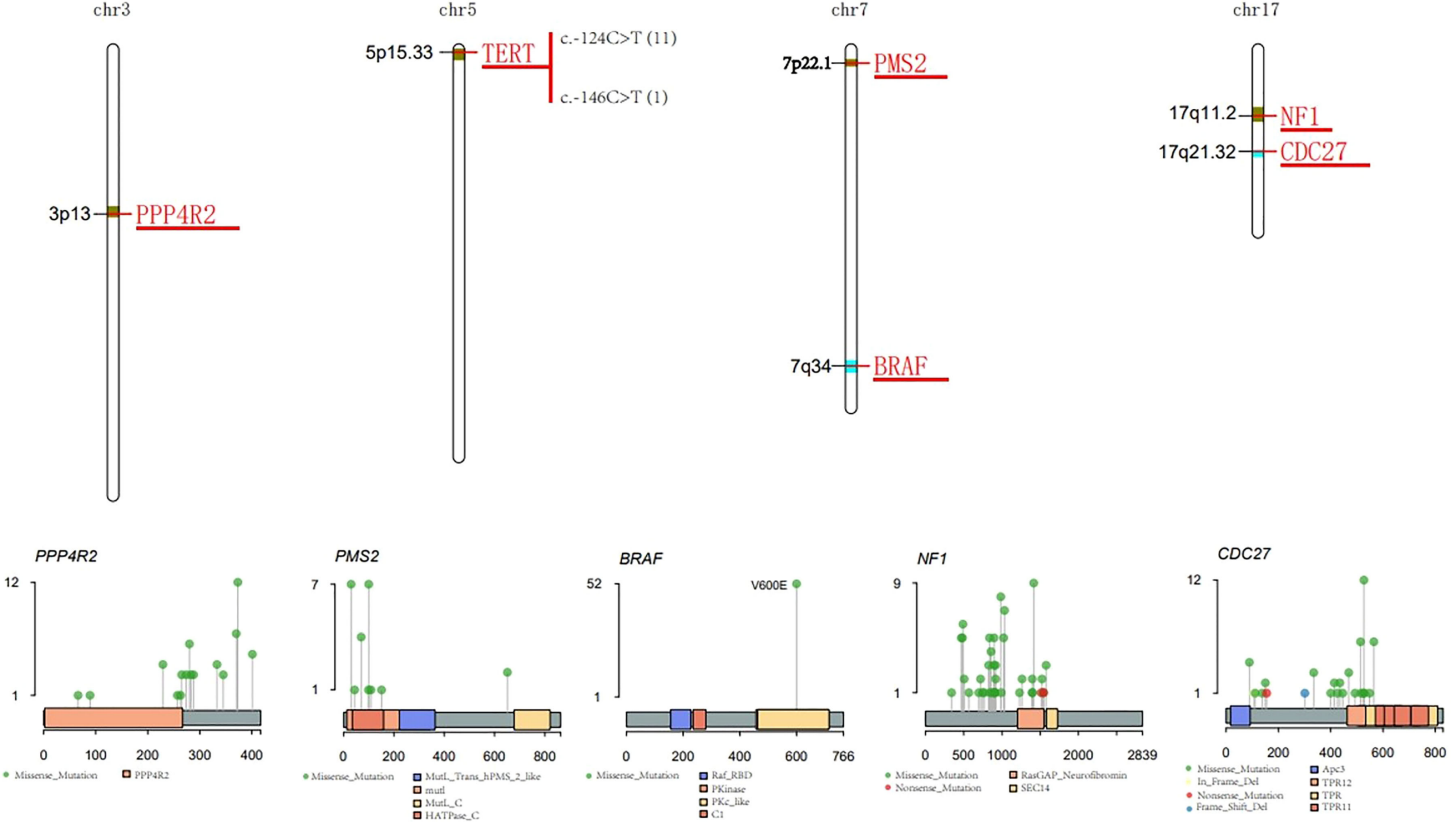
Characteristic of major mutant genes. Characteristic of BRAF, CDC27, NF1, PPP4R2, TERT and PMS2 gene.

BRAF is known to play important regulatory roles in the proliferation and differentiation of thyroid cancer. In this study, V600E was the only observed variation in BRAF gene -mutated patients (**Figure 3**).

CDC27 is involved in the regulation of cell mitosis, and is generally highly expressed in thyroid cancer. The I527Q mutation that occurs in the tetratricopeptide repeat (TPR) protein domain accounted for the highest proportion of patients (**Figure 3**).

The NF1 gene encodes neurofibromin, which negatively regulates the RAS/MEK pathway and helps control cell growth, differentiation and survival. Mutations in the NF1 gene may lead to dysregulation of the RAS-RAF-MEK-ERK signaling pathway, which may cause cells to grow, divide, and replicate in an uncontrolled manner, and may lead to tumor growth. NF1 gene mutations were detected in 14% of patients, most of which were missense mutations (**Figure 3**).

PMS2 is a DNA mismatch repair (MMR) protein. Members of the PMS2 gene family are located in clusters on chromosome 7. DNA MMR defects promote the genomic instability of the subgroup of PTC. In total, 13% of the 83 PTC patients carried the PMS2 missense mutation (**Figure 3**).

**Figure 4:**
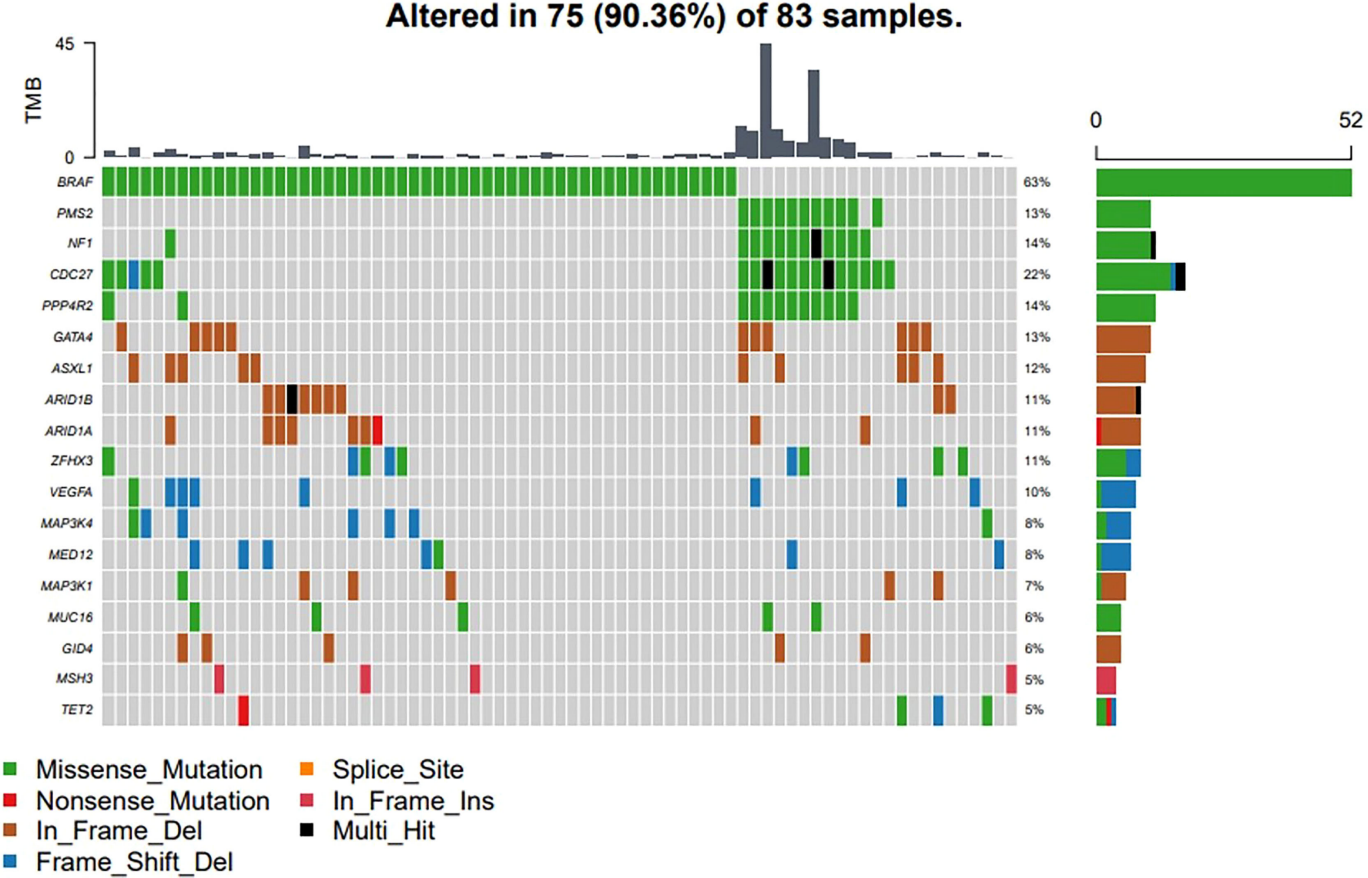
Driver genes and clinical relevance. The driver events were classified into two major categories, the BRAF induced RAS-RAF-MEK-ERK signaling pathway, and the NF1 induced dysregulation of the RAS-RAF-MEK-ERK signaling pathway.

Telomerase reverse transcriptase (TERT) is the catalytic subunit of telomerase, located on chromosome 5p15.33. The two most common mutation sites in the TERT promoter region are C228T and C250T. These two mutations can enhance the transcription of the TERT promoter. In 83 PTC patients, 11 patients had the C228T (c.-124C>T) mutation, and 1 patient had the C250T (c.-146C>T) mutation (**Figure 3**). There was a significant correlation between BRAF and TERT promoter mutations (p<0.05) (**Figure 4**).

PPP4R2 participates in related pathways including DNA double-strand break repair. In total, 14% of PTC patients had this mutation, and the main type of mutation was the missense mutation (**Figure 3**).

The RAS gene regulates the MAPK and PI3K/Akt pathways during the occurrence and development of thyroid cancer. The NRAS family mutations were present in 3 patients, including 1 missense point mutation and 2 copy number gains.

### Classification of Driver Mechanisms and Clinical Relevance

Aiming to explore the underlying driver mechanisms and their clinical significance, studies have reported that the proportion of driver genes in thyroid cancer is the lowest compared with other cancers. However, we found that 90.36% (75/83) of patients carried at least one mutation on known driver genes from a known driver gene list (**Figure 4**), which means that the genetic factors in a large proportion of the PTC cohort are still unknown. To explore the underlying driving mechanism for more patients, we employed a machine learning based model, DriverML (7), to predict the driving patterns and identify novel driver genes in the 83 PTC patients. The driver events were classified into two major categories, the BRAF induced RAS-RAF-MEK-ERK signaling pathway, and the NF1 induced dysregulation of the RAS-RAF-MEK-ERK signaling pathway. For 8 PTC cases, DriverML did not find any driver mutations.

**Figure 5:**
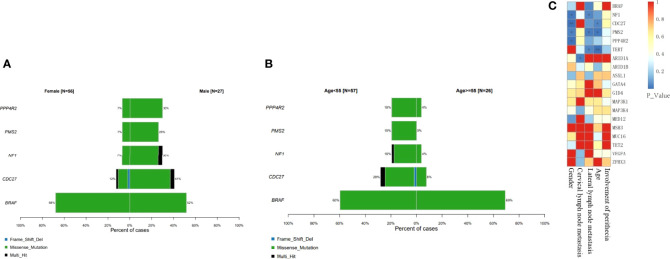
Classification of driver mechanisms and clinical relevance. BRAF gene mutations always occur in female patients and in patients over 55 years of age (P > 0.05). Mutations of NF1, PMS2 and TERT were all related to a high rate of lateral cervical lymph node metastasis. **(A)** Proportion of different gene mutations in different gender groups. **(B)** Proportion of different gene mutations age groups. **(C)** Correlation between single mutant gene clinical manifestation (*P<0.05; **P<0.01).

BRAF gene mutations may occur in all age groups, especially in female patients (**Figure 5A**) and in patients over 55 years of age (**Figure 5B**). TERT mutations usually occur with BRAF mutations in older patients, and the proportion of female patients was significantly higher (72.73%, 8/11). The Cdc27, NF1, PMS2 and PPP4R2 gene mutations were more common in men (P<0.05). In addition, CDC27 and PMS2 mutations were more common in patients under 55 of age (P<0.05), but on the other hand, TERT mutations were more common in patients over 55 years of age (P<0.05) (**Figure 5B**). Mutations of NF1, PMS2 and TERT were all related to a high rate of lateral cervical lymph node metastasis (P<0.05) (**Figure 5C**).

**Figure 6:**
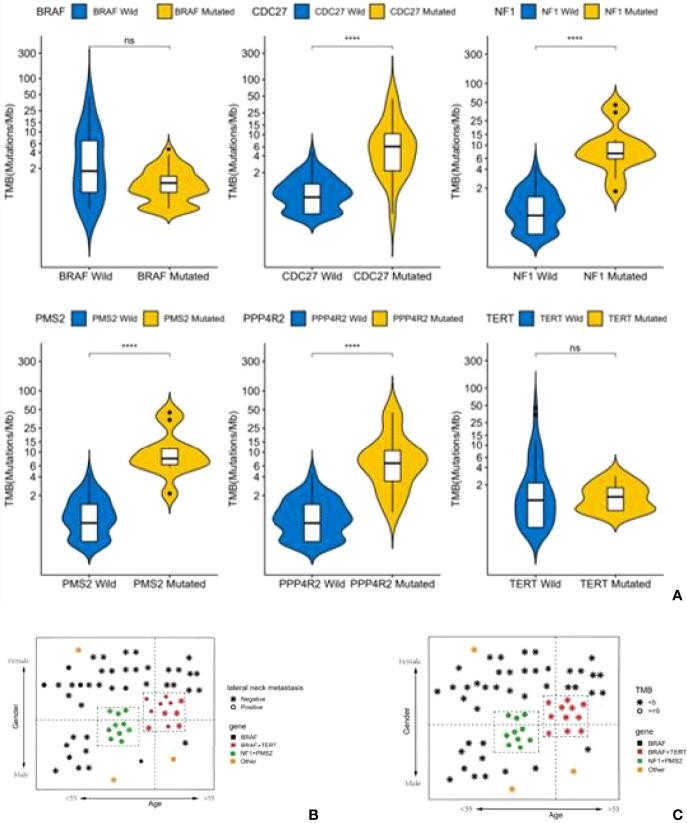
A high tumor mutational burden (TMB) in patients with mutations of NF1, PMS2, CDC27 or PPP4R2. Patients with lateral cervical lymph node metastasis were more common in both the BRAF-TERT mutation group and the NF1-PMS2 group. **(A)** Difference of TMB level between single gene mutant and wild type (ns,no significance,****:*P*<0.001). **(B)** Status of lateral cervical lymph nodes in people patients with different gene mutations. **(C)** The level of TMB in patients with different gene mutations.

At the same time, the TMB values for these patients with mutations of NF1, PMS2, CDC27and PPP4R2 were higher than for other patients (cut-off value=5) (P<0.001). In addition, patients with lateral cervical lymph node metastasis were more common in both the BRAF-TERT mutation group and the NF1-PMS2 group (**Figure 6A**). The results shows 14% (12 cases) of patients had TERT promoter mutations, which further led to an increase in the rate of lateral cervical lymph node metastasis, and 11 of these patients had BRAF mutations at the same time. The risk in the BRAF+TERT mutation group (lateral cervical lymph node metastasis rate 54.5%, 6/11) is higher in old-females while NF1+PMS2 mutation group mostly happened in young-males of the lateral cervical lymph node metastasis. (**Figure 6B**). There also shows that the TMB index in the NF1 + PMS2 group was significantly higher than in the BRAF+TERT group (even the BRAF group) (**Figure 6C**).

## Discussion

Transduction of multiple signaling pathways in PTC is closely related to a series of oncogenic processes such as tumor cell proliferation and apoptosis. Understanding the signaling pathways involved in its occurrence and development is of great significance for in-depth study of thyroid cancer. Dysfunctions in signaling pathways might be used as biomarkers in the diagnosis and prognosis of thyroid cancer. At present, multiple signaling pathways involved in thyroid hormones include mitogen activated protein (MAPK), phosphatidylinositol 3-kinase (PI3K)/protein kinase B (PKB, or AKT), and calcium and mTOR pathways. Continuous activation and interaction between multiple signaling pathways can induce the occurrence, development and metastasis of thyroid cancer (8).

### Main Driving Mutations and Related Mechanisms of Carcinogenesis in PTC

The incidence rate of PTC has increased year by year, and has been developing in all age groups. Especially in recent years, there is a trend for younger onset. BRAF gene is the mutated gene in most thyroid cancers, but the clinical manifestations of some thyroid cancers are definitely different. Using a computer algorithm to identify major driver mechanisms in our PTC cohort, the results showed that, in addition to BRAF mutations, NF1, PMS2, CDC27 and PPP4R2 gene mutations are also involved in the development of thyroid cancer, and joint mutations often occur. DriverML predicted that BRAF and NF1/PMS2 mutations are the main driving mutations in thyroid cancer, and they play a certain role in regulating its development. Because BRAF and NF1/PMS2 have significant mutually exclusive relationship, we speculate that the mechanism of gene mutation leading to the occurrence and development of thyroid cancer may be different between the two groups. However, the functions of NF1/PMS2 remained to be uncovered in PTC oncogenesis.

The BRAF V600E point mutation can activate BRAF kinase to cause carcinogenesis. When a BRAF gene mutation activates the downstream MEK->ERK-MAPK signaling pathway and downstream signaling molecules to enhance the expression of proto-oncogenes, it has a certain effect on the development and metastasis of thyroid cancer (9–11). The incidence rate of the BRAF V600E point mutation in our study was similar to that in the PTC cohort reported in The Cancer Genome Atlas (TCGA) (12).

The mechanism of association between NF1 mutation and PTC is not clear. In previous reports, NF1 mutation is mostly considered to be related to anaplastic and undifferentiated thyroid cancer (13). In PTC tissue, the positive detection rate of NF1 is not high, so it is often easily ignored (12). In fact, the loss of function caused by NF1 gene mutation may prolong the activation time of the Ras dependent signal pathway, and, in addition, may cause abnormal signal expression which is different from the classic BRAF mutation, resulting in tumor dedifferentiation (14).

PMS2 and PPP4R2 are DNA mismatch repair related genes. Studies have shown that the functional defect of DNA mismatch repair is most common in high mutation load thyroid cancer (46%), especially in ATC, and there is a lack of typical BRAF, RAS or RET thyroid oncogene mutations (15). Our results show that PMS2 mutations often occur simultaneously with NF1 mutations, which may indicate the dedifferentiation tendency and high mutation load of this group of tumor cells. Further research is needed to understand the role of mismatch repair gene defects in thyroid tumors, as they could predict the response of solid tumors to PD-1 blockade (16).

CDC27 is the core subunit of APC/C (encoding protein APC/C is a multi-subunit E3 ubiquitin ligase subtype) that can activate APC/C to recognize and degrade target substrates (17). At the same time, CDC27 has a relatively high mutation rate in multiple cancers. Detrimental mutations in CDC27 may weaken the activity of APC/C and cause the accumulation of the target substrate, or it may reduce the error rate of chromosome segregation by extending the mitosis (18). The accumulation of substrates and prolonged mitosis may play an important role in cell proliferation and promote the development of thyroid cancer.

The second highest mutation gene of PTC in TCGA data is RAS, which is mostly considered to be related to tumor invasiveness. Only 3 cases of NRAS mutations were found in our study, which may be due to the inclusion of more cases with lateral cervical lymph node metastasis, and the exclusion of 17 small samples. In addition, an analysis of 355 Chinese people also showed that the proportion of RAS mutation was only 2.8% (19). At the same time the proportion of fusion mutations is as high as 6.03%-13.8% (19, 20), which is similar to our results. These results may be related to population differences.

### Different Driver Genes and Clinical Risk Stratification

Although BRAF mutation accounts for the majority in PTC population, it is difficult to explain the clinic polymorphism. Some researchers used next-generation sequencing to screen high-risk genes in Chinese PTC population. It was found that RBL2, FOXO1, MUC6, PCDHB9, NOTCH1, FIZ1, and RTN1RAS mutations were related to event free survival (21). Other studies have found some markers that may affect the prognosis from the comparison of transcriptomics (22). However, due to the good prognosis and diversified genetic characteristics of PTC, these indicators may be difficult for doctors to analyze the clinical characteristics of patients. Therefore, we analyzed the sequencing samples from another idea. Instead of establishing a model to explain all types of PTC, we expected to find a class of specific mutations with the same clinical characteristics to guide the clinical individualized treatment and research.

Clinically, lateral cervical lymph node metastasis is an important indicator of tumor risk stratification and active iodine therapy. Our study showed that patients with the most common mutation of BRAF had a lateral lymph node metastasis rate of 22.2%. Studies have shown that, although the overall lymph node metastasis rate of patients with BRAF mutations is high (23), most are micro metastases in the central region, and may not affect the long-term prognosis (24, 25). Therefore, it is necessary to group PTC patients in more detail according to different mutant genes to guide treatment and judge prognosis.

Mutations in the TERT gene promoter are among the most reported high-risk mutations, and are strongly related to tumor dedifferentiation and metastasis (26). In this study, 14% (12 cases) of patients had TERT promoter mutations, which further led to an increase in the rate of lateral cervical lymph node metastasis, and 11 of these patients had BRAF mutations at the same time. Therefore, from a clinical point of view, the risk in the BRAF+TERT mutation group (lateral cervical lymph node metastasis rate 54.5%, 6/11) is higher than in the BRAF-TERT mutation group (14.6%, 6/41). From this, we may conclude that patients with BRAF mutations should be classified as a high-risk group when they also have TERT promoter mutations.

Another high-risk group for PTC was also found in our study. PTC in this second group is often not caused by BRAF mutations, but by NF1 mutations (usually combined with PMS2 mutations). The lateral lymph node metastasis rate in this group of patients is also high (70%, 7/10), and they can be classified as a high-risk group clinically, but other clinical characteristics are significantly different from the BRAF+TERT group of patients (**Figure 6B**). Patients in the NF1 mutation group are mostly young men under the age of 50 years, which may also be the reason why young men tend to have a higher degree of malignancy in the clinic.

It is interesting to note that the TMB index in the NF1 + PMS2 group was significantly higher than in the BRAF group (even the BRAF + TERT group). TMB is an indication for cumulated mutations in the cancer genome. A number of studies in non-small cell lung cancer (NSCLC) and melanoma have shown that TMB is related to the clinical benefit of patients, therefore can be used as an independent predictor to distinguish the population which may benefit from immunotherapy in certain cancer types (27, 28). The TMB distribution of PTC patients ranks in the lower spectrum in pan-cancer statistics (29). It is reported that PTC patients with a high tumor mutation burden may have a poor prognosis (30). However, TMB distributions in Chinese PTC patients have not been reported. In our study, more than 90% of patients with thyroid cancer have a TMB lower than 10, which is consistent with the TMB distribution in PTC patients in other studies (29, 31). However, when grouped by driver genes, we found that the TMB values in the NF1 group were higher, which was significantly different from the BRAF+/-TERT group. The NF1 group is often combined with PMS2 and PPP4R2 mutations, which are both related to DNA damage repair. DNA mismatch repair gene mutations in the NF1 group may lead to accumulation of insertion-deletion mutations in the genome and a high level of TMB.

## Conclusion

Targeted sequencing in Chinese patients with PTC using the 688-gene O-seq panel is a valuable method for the molecular classification of these patients. In addition, we found that BRAF and NF1 mutations are two of the main regulators in Chinese PTC patients, and they play critical but different roles in regulating the development of thyroid cancer. Furthermore, we identified two groups of high-risk PTC patients with different clinical characteristics.

## Data Availability Statement

The original contributions presented in the study are included in the article/supplementary material. Further inquiries can be directed to the corresponding authors.

## Ethics Statement

The studies involving human participants were reviewed and approved by Beijing Tongren Hospital. The patients/participants provided their written informed consent to participate in this study.

## Author Contributions

WG, JH have contributed equally to this work. WG, JH, ZH, and XC contributed to conception and design of the study. TS and HD organized the database. WG and JH performed the statistical analysis. WG and JH wrote the first draft of the manuscript. WG, JH, HD, and TS wrote sections of the manuscript. All authors contributed to manuscript revision, read, and approved the submitted version.

## Conflict of Interest

The authors declare that the research was conducted in the absence of any commercial or financial relationships that could be construed as a potential conflict of interest.

## Publisher’s Note

All claims expressed in this article are solely those of the authors and do not necessarily represent those of their affiliated organizations, or those of the publisher, the editors and the reviewers. Any product that may be evaluated in this article, or claim that may be made by its manufacturer, is not guaranteed or endorsed by the publisher.
